# Considerations for ethics review of big data health research: A scoping review

**DOI:** 10.1371/journal.pone.0204937

**Published:** 2018-10-11

**Authors:** Marcello Ienca, Agata Ferretti, Samia Hurst, Milo Puhan, Christian Lovis, Effy Vayena

**Affiliations:** 1 Health Ethics and Policy Laboratory, Department of Health Sciences and Technology, ETH Zurich, Zurich, Switzerland; 2 Institute for Ethics, History and the Humanities, Faculty of Medicine, University of Geneva, Geneva, Switzerland; 3 Epidemiology, Biostatistics and Prevention Institute, University of Zurich, Zurich, Switzerland; 4 Division of Medical Information Sciences, Department of Radiology and Medical Informatics, University Hospital of Geneva, Geneva, Switzerland; Paediatric Centre of Excellence, ZAMBIA

## Abstract

Big data trends in biomedical and health research enable large-scale and multi-dimensional aggregation and analysis of heterogeneous data sources, which could ultimately result in preventive, diagnostic and therapeutic benefit. The methodological novelty and computational complexity of big data health research raises novel challenges for ethics review. In this study, we conducted a scoping review of the literature using five databases to identify and map the major challenges of health-related big data for Ethics Review Committees (ERCs) or analogous institutional review boards. A total of 1093 publications were initially identified, 263 of which were included in the final synthesis after abstract and full-text screening performed independently by two researchers. Both a descriptive numerical summary and a thematic analysis were performed on the full-texts of all articles included in the synthesis. Our findings suggest that while big data trends in biomedicine hold the potential for advancing clinical research, improving prevention and optimizing healthcare delivery, yet several epistemic, scientific and normative challenges need careful consideration. These challenges have relevance for both the composition of ERCs and the evaluation criteria that should be employed by ERC members when assessing the methodological and ethical viability of health-related big data studies. Based on this analysis, we provide some preliminary recommendations on how ERCs could adaptively respond to those challenges. This exploration is designed to synthesize useful information for researchers, ERCs and relevant institutional bodies involved in the conduction and/or assessment of health-related big data research.

## Introduction

The generation of digital data has drastically increased in the last years due to the ubiquitous deployment of digital technology as well as advanced computational analytics techniques [1, 2]. The term big data is still vaguely defined. In general terms, big data involves large sets of data with diverse levels of analysable structuration, coming from heterogeneous sources (online data, social media profiles, financial records, self-tracked parameters, etc.), produced with high frequency and which can be further processed and analysed using computational techniques. While the term big data has become nearly ubiquitous, there is controversy over what data volumes are sufficiently large to obtain the big data label. Dumbill, for example, suggested that data should be considered big when they cross the threshold of the conventional databases systems’ capacity in processing information [3].

Big data trends characterize various sectors including basic science [1, 4], business [5], government [6], national security [7] and transportation [8]. Big data trends have increasingly pervaded also the healthcare domain, as new health-related data sources have grown in volume and variety, and became available for large-scale aggregation and high-speed analysis [9]. These include Electronic Health Records (EHRs), data from mobile health (mHealth) applications, medical blogs and web-networks [10] [11], healthcare robotics [12], medical internet of things [13], as well as direct-to-consumer genetic [14], and screening tests [15]. Additionally, health-related information can be derived not only from digital health applications, but also from non-strictly-medical data sources [16] such as online personal dietary programs, fitness club memberships and Twitter hashtags [17]. Health-related big data is the umbrella term used to describe extremely large and heterogeneous data sets that may be analysed computationally to reveal patterns, trends, and correlations, that have relevance for human health [18].

The availability of health-related big data holds the promise of exerting a positive impact on biomedical research. For example, tailoring diagnostics to automated analyses of high resolution images has become a standard procedure in cancer research [19]. In parallel, mapping and collecting large-scale data volumes enables the creation of epidemiological models that can inform about an epidemics’ space-time propagation. Finally, novel and patient-tailored therapeutic opportunities might emerge from the possibility of continuously monitoring patient health, tracking pathologic characteristics at specific points in time, and aggregating heterogeneous data sources [20]. These benefits might occur both in public health and at the individual level. Bates [21] argued that the use of big data has a valuable impact on public health, since it might help identify and promptly intervene on high-risk and high-cost patients.

While opening the prospect of clinical benefit, the use of health-related big data raises important challenges. In light of their methodological novelty, potentially far-reaching impacts, and computational complexity, big data approaches to human health have been claimed to raise ethical, legal and social implications [22]. Ethical and legal challenges include the risk to compromise privacy, personal autonomy, and the solidarity-based approach to healthcare funding, as well as effects on public demand for transparency, trust, and fairness while using big data [23]. Furthermore, authors have listed data heterogeneity, data protection, analytical flows in analysing data, and the lack of appropriate infrastructures for data storage as critical technical and infrastructural issues that might endanger a big-data-driven healthcare [24]. While some of these challenges have received scientific and institutional attention, other ones have remained largely unexplored. In 2016, a review identified a number of areas of concern associated with health-related big data that did not obtained adequate attention among researchers [22]. These included group-level ethical harms, the intimate link between epistemological and ethical issues, the distinction between harms to data subject resulting from, respectively, academic and commercial uses of big data, the problematic fiduciary relationship between data custodian and data subjects, the role of data ownership and intellectual property as a mechanism for data control, and, finally, the provision of data access rights to data subjects.

The ethical, legal and social implications of health-related big data raise novel challenges also for Ethics Review Committees (ERCs). ERCs and institutional review boards are increasingly requested to evaluate an ever-growing number of research projects and associated activities involving big data (large data volumes and big data analytics), whose risks and benefits often appear hard to assess. Some authors have called for the development of comprehensive regulatory policies for healthcare entities and new computing safeguards that can address public concerns, such as the protection of individually identifiable information [25]. However, in absence of specific guidelines and comprehensive evaluation studies, ERCs might be facing uncertainty on how to review health-related big data projects and according to which evaluative criteria. In fact, researchers have observed that traditional conceptual tools and/or legal requirements for ethics review in clinical research like informed consent, minimal risk and fair subject selection might be of limited help, if not ill suited, for the evaluation of big data projects [26, 27]. The reason for that stems from the fact that these tools were conceived in the context of conventional clinical research (e.g. clinical trials) not in connection to the evolving applications and innovative research designs of big data research [27]. For example, informed consent is often not practical to obtain for studies involving a retrospective access to data from millions of individuals.

The nature of big data studies also challenges the current mandate and purview of ERCs. For example, studies involving publicly available and anonymized data have traditionally been perceived to be outside of the purview of ERCs. This would include data from Twitter (which are public by default), Facebook or other online platforms. Furthermore, ethical safeguards for human subjects research “are often written with definitions that exclude Internet research”[28]. This is problematic for a twofold reason. First, research has shown that big data analytics can reveal sensitive information from seemingly innocuous public data points, including information that the original data generators might reasonably wish to keep private. For example, a recent study has successfully used deep neural networks to predict the sexual orientation of users based on facial images from public profiles posted on dating website [29]. Second, several studies have shown that de-identified [30] and even anonymized data [31] can be reverse engineered to re-identify individuals, leading experts to the conclusion that “there is no such thing as anonymous data”. This raises the question of whether big data projects should require oversight by an ERC even when the data collected are public and anonymized or de-identified. A recent systematic review has concluded that most normative documents deem the review of an ERC as *necessary* to address the concerns associated with the use of anonymized data for research [32]. In contrast, when ERCs waived the review of big data studies involving publicly available and anonymized data repositories because they considered them outside their purview, such as in the case of Facebook’s “emotional contagion” study [33], experts criticized this narrow interpretation of the ERC’s mandate [34].

In the present study, we aim to identify the promises and challenges of health-related big data research that have relevance for ERCs. Furthermore, we use these findings to suggest how ERCs could adaptively respond to this methodological transformation. This exploration is designed to synthesize useful information for researchers, ERCs and relevant institutional bodies involved in the conduction and/or assessment of health-related big data research.

## Methods

On the 18^th^of September 2018 we conducted a scoping review of the scientific literature and searched five databases (EMBASE, Web of Science, Pubmed, IEEE Xplore, and Scopus) to retrieve eligible publications. We searched title, abstract, and keywords for the terms: ("big data" OR “Artificial Intelligence” OR "data science" OR "digital data") AND (“medical” OR “healthcare” OR “clinical” OR "personalised medicine") AND (“policy” OR “ethics” OR “governance” OR "ethics committee" OR “IRB” OR "review board" OR “assessment”). Query logic was modified to adapt to the language used by each engine or database. Screening identified 1093 entries. All entries were imported into the Endnote literature manager software. Three phases of filtering were performed independently by two researchers to minimize subjective bias.

**Fig 1:**
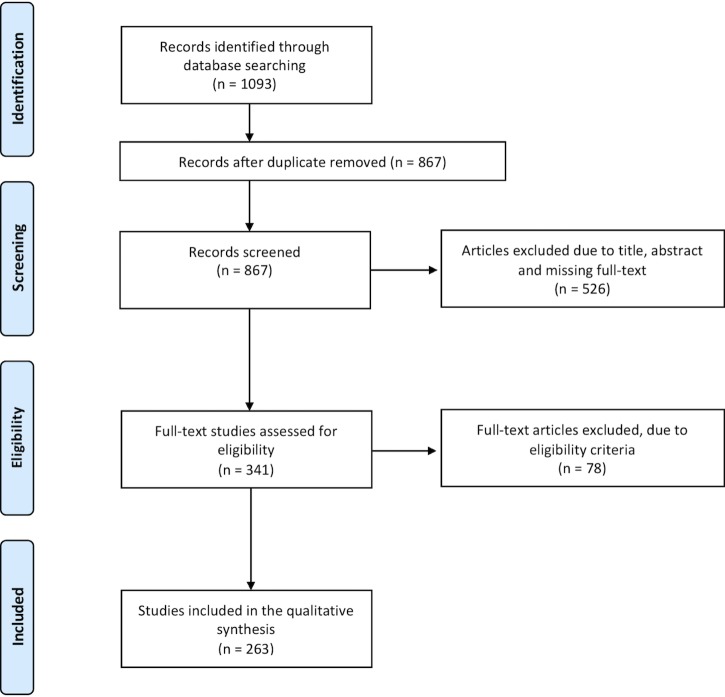
Scoping literature review flow chart (PRISMA).

The scoping review is a review method aimed at synthesizing research evidence and mapping the existing literature in a certain field of interest [35]. Unlike a systematic review, scoping review methods are considered of particular use when the topic has not yet been extensively reviewed or is of a complex or heterogeneous nature [35, 36]. Following the recommendations by Pham et al. [36], the study selection process was conducted and presented using the Preferred Reporting Items for Systematic Reviews and Meta-Analyses (http://prisma-statement.org/) as a guide (see Fig 1).

First, duplicates were removed both automatically using the Endnote tool for duplicate detection and manually based on abstract screening. A total of 226 articles was removed at this stage.

Second, eligibility assessment was performed independently by two of the co-authors on the remaining 867 articles through title-abstract screening and, subsequently, full text screening. Diverging inclusion choices between the two reviewers were discussed with the research group with documented reasons. Studies included in the synthesis had the following features: (i) original articles, book chapters or conference proceedings; (ii) written in English, Italian, French or German (languages spoken by the researchers); (iii) published before September 18^th^, 2017; and (iv) focused on the assessment of big data trends in the biomedical/healthcare context. Reviews, letters to the editors, business reports and dissertations were not included. A total of 263 studies were included in the final synthesis and imported manually into Microsoft Excel 15.40 format based on a shared data-charting form. Following the recommendations to enhance scoping study methodology delineated by Levac et al. [37], the data-charting form was collectively developed by our research team to determine which variables to extract from the review data.

Third, based on the same recommendations, we performed both a descriptive numerical summary and a thematic analysis. In the former analysis, both relative and cumulative frequencies were extracted and graphically represented using bar charts. Following Arksey and O'Malley [36], our descriptive numerical summary also included the total number of articles included, types of study design (empirical vs. non empirical), years of publication etc. In the latter analysis, recurrent thematic patterns were identified through full-text screening and subsequent coding. The coding phases was independently performed by two researchers. Once conceptually stable thematic patterns emerged from the codes, these were grouped together into a system of themes and subthemes. All entries were checked anew through an automated text search for the presence of the emerging themes. Following Braun and Clarke [38], codes that did not seem to fit into any main theme, were temporarily housed in a “miscellaneous” group and subsequently either clustered into a new theme or reallocated to an existing thematic group after consultation. Internal consultation was performed among all members of our research team to integrate and validate our findings.

## Results

**Fig 2:**
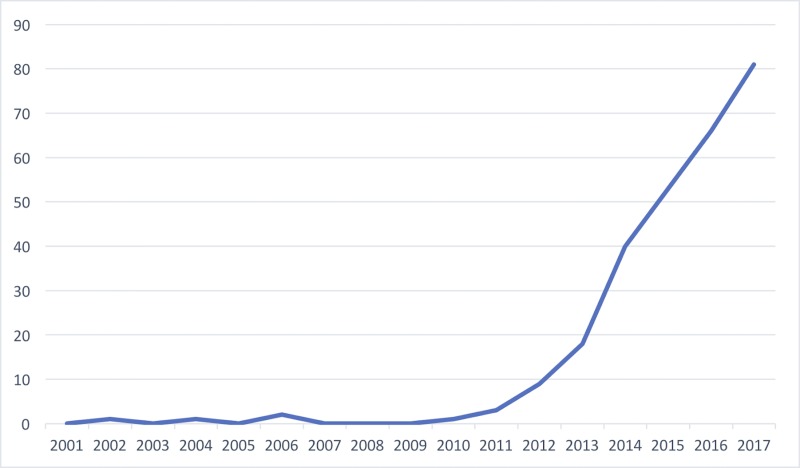
Increase over time in research papers discussing the challenges of health-related big data. N.B. The search was performed on September 18, 2017. Therefore, the full number of articles for year 2017 was calculated by projecting the data until September 18.

Our results reveal a large, diverse and rapidly growing body of literature on the impact of big data in the biomedical domain. Data show that the overall number of articles published in the time period 2012–2017 is 131 times higher compared to the period 2001–2005 as represented in Fig 2.

Data breakdown by medical speciality and field of medical application indicates that big data approaches have been discussed and evaluated in relation to several branches of medicine including neurology and psychiatry (n = 31), oncology (n = 17), cardiology (n = 8), medical genetics (n = 8), immunology and infectious diseases (n = 8), as well as nuclear medicine and radiology (n = 6). Crossfield evaluations of health-related big data appeared highly prevalent (n = 155).

Thematic analysis identified a number of potential opportunities and challenges associated with health-related big data approaches, many of which have relevance for ethics review. Opportunities could be grouped into four main themes: biomedical research, prevention, healthcare delivery and healthcare management. Potential benefits in the research domain include the possibility of collecting real-world data, accelerating the development of new medical technology and facilitating translational research. Big data was also associated with the improvement of preventive measures at both the individual and population level. In relation to care delivery, the following benefits were envisioned: precision and personalized medicine, earlier and more accurate diagnostics, enhanced clinical decision-making, ubiquitous health monitoring, improved patient safety and better therapy. Subsequent numeric analysis of thematic clusters is presented in Table 1.

**Table 1:** Recurrent promises and challenges associated with health related big data that have relevance for ethics review.

Opportunities	Challenges
**Healthcare Delivery (n = 276)**	Technical (n = 125)
**Healthcare Management (n = 90)**	Ethical (n = 81)
**Biomedical Research (n = 85)**	Methodological (n = 66)
**Prevention (n = 45)**	Regulatory (n = 39)
	Social (n = 16)
	Infrastructural (n = 11)
	Financial (n = 10)

N.B. The same study might describe >1 promise or challenge.

Envisioned challenges appeared of seven major types: technical (n = 125), ethical (n = 81), methodological (n = 66), regulatory (n = 39), social (n = 16), infrastructural (n = 11) and financial (n = 10). Technical challenges relate to issues inherent in the data ecosystem. These include data security, data quality, data storage, data linkage, and tools for data reuse. Methodological challenges relate to the system of methods used in the study and include issues of standardizing data and metadata, integrating and processing data, monitoring resource utilisation and compensating for incomplete data. Regulatory challenges relate to rules or directives such as those regulating data ownership and the accountability of actors in relation to the potential risks associated with using and managing data. Social challenges are those that have relevance for human society and its members. These include, among others, secondary uses of data in relation to participants consent, sociocultural and ethnic bias and subsequent risk of discrimination, power asymmetries between data subjects and data controllers. Finally, financial and infrastructural issues included the financial viability of data storage sites and to the level of preparedness of existing infrastructures respectively.

**Fig 3:**
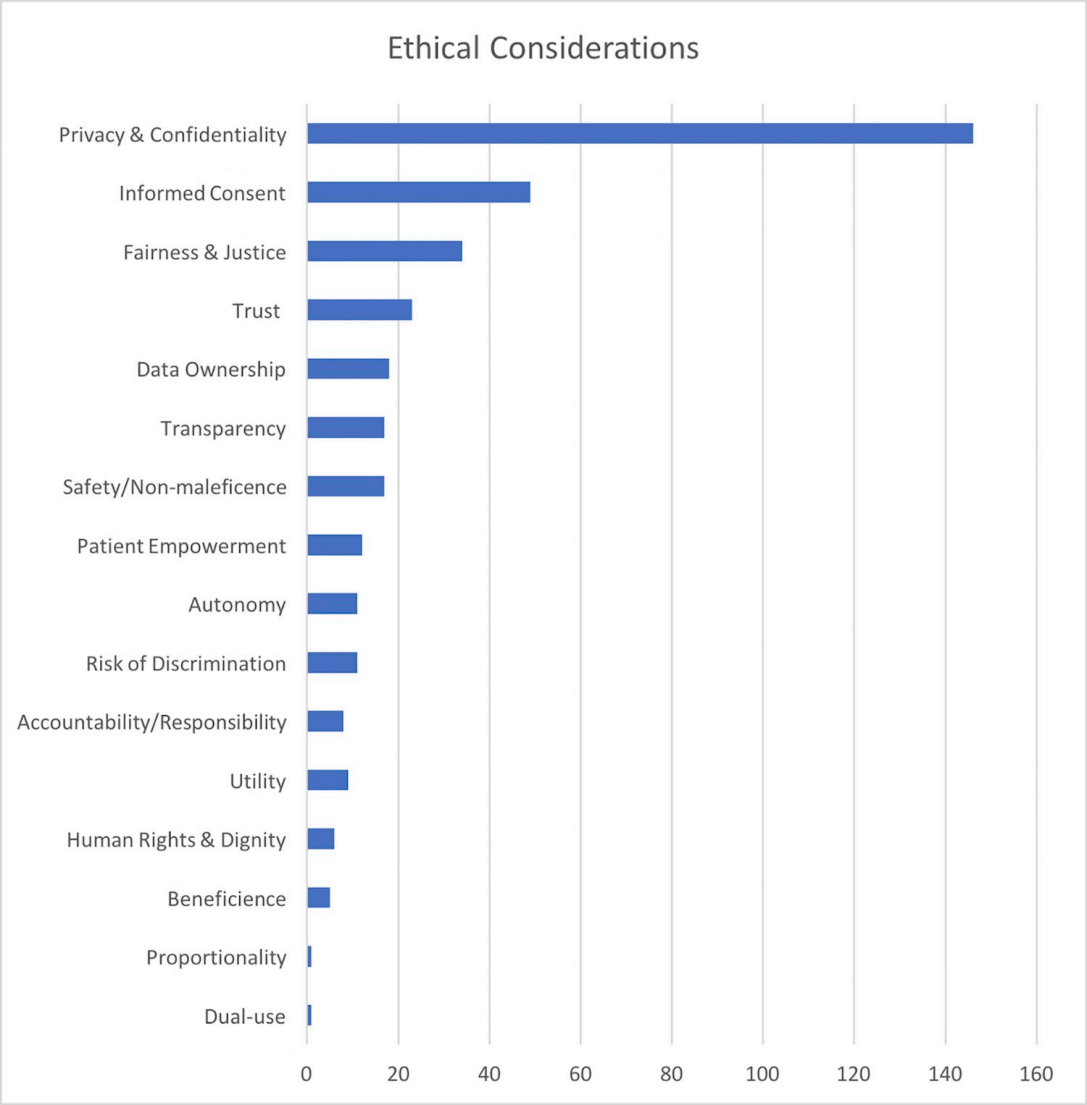
Frequency of ethical considerations associated with health-related big data studies.

Ethical challenges are those related to moral principles. Our analysis revealed privacy and confidentiality to be by far the dominant concern (n = 146) in the ethical domain, followed by informed consent (n = 49), fairness and justice (n = 34), trust (n = 23), data ownership (n = 18) and others. Fig 3 presents a full overview of ethical considerations associated with health-related big data studies with associated relative frequencies.

While the analysis revealed a number of implications with relevance for ethics review, only 13% of reviewed studies provided specific normative recommendations for ERCs or other institutional review boards. Data breakdown by study methodology revealed that only a small portion of those recommendations (n = 5; 14%) was informed by empirical methods.

**Fig 4:**
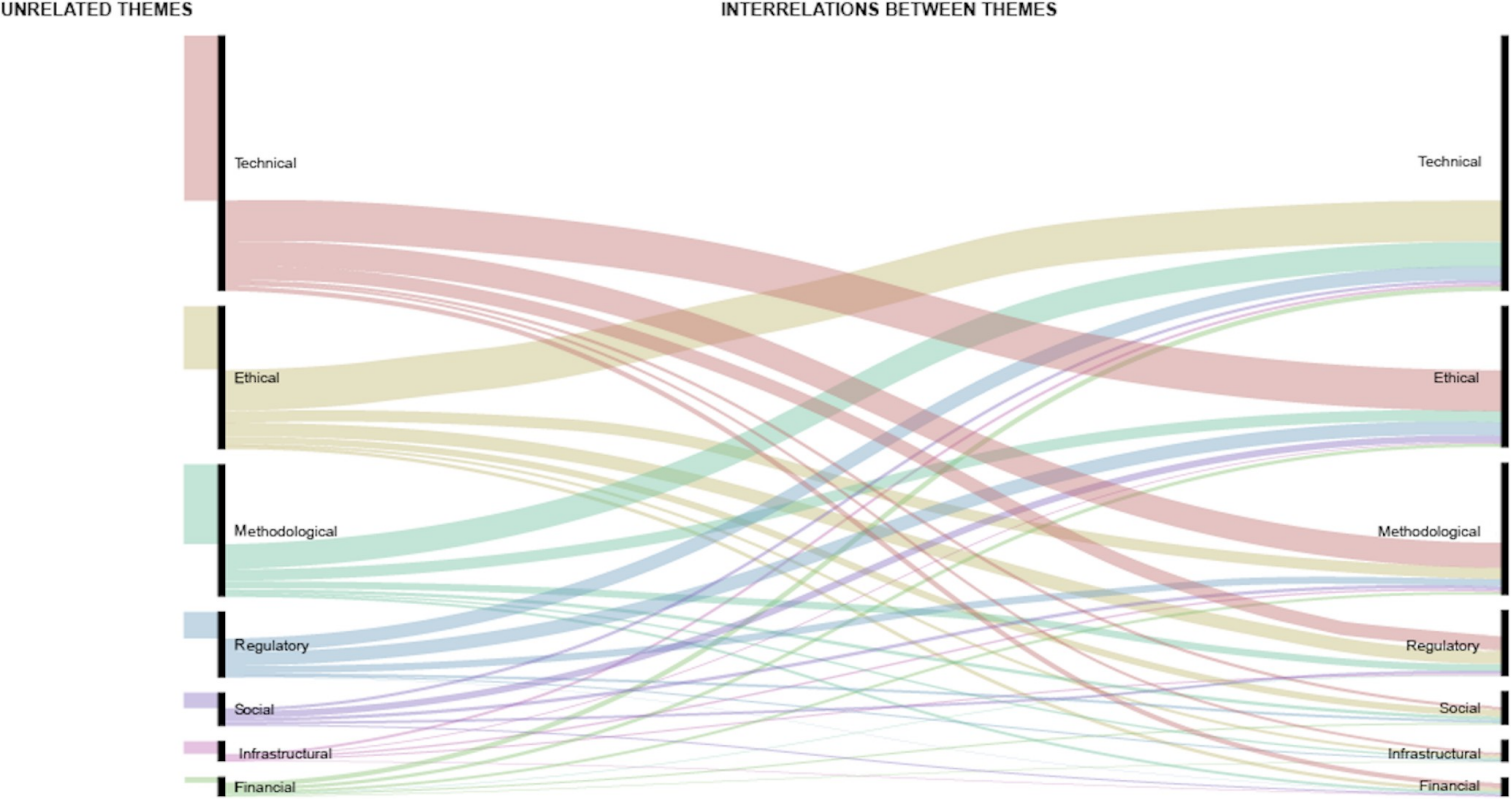
Alluvial diagram of mutual interrelations between different thematic families (figure credit Joanna Sleigh).

A subsequent analysis of thematic co-occurrences shows a strong mutual relationship between different thematic families, especially between technical and ethical issues, as shown in Fig 4. In particular, technical issues such as data security and data linkage were often presented in coordination with ethical issues such as personal privacy.

## Limitations

This study presents four main limitations. First, a selection bias might be present since the search retrieved only articles written in languages known by the researchers (English, French, German and Italian), excluding articles written in other languages. A similar limitation affects database selection as searching other databases may have possibly identified additional relevant studies. While this risk of selection bias applies to any review since the number of databases that can be feasibly searched is always finite, we attempted to minimize selection bias by exploring both domain-general and domain-specific databases, including the major databases in biomedical research and computer science, which represent the primary interdisciplinary intersection when it comes to biomedical big data. Second, as it was often observed in relation to scoping reviews, the explorative nature and broad focus of our search methodology makes it ‘unrealistic to retrieve and screen all the relevant literature’ [39]. However, one advantage of the scoping methodology is the opportunity to explore also the grey literature and the secondary sources (e.g. bibliographies of retrieved papers), which is likely to increase comprehensiveness. The breadth of the research focus might have inevitably affected the depth of the analysis. The reason for that stems from the fact that the outcomes of a scoping review, compared to systematic review methods, are “more narrative in nature” [40] and usually not presented through descriptive statistical analysis. Finally, our review included very heterogeneous studies and did not assess the study quality. The reason for that stems from the fact that our main goal was to explore the entire range of challenges that have relevance for ERCs, regardless of how those challenges were originally addressed and discussed. While these four limitations might prevent generalization, we believe that the scoping methodology was best suited to reflect the explorative nature and broad focus of our research question. In fact, it has often been noted, that scoping reviews are not intended to be exhaustive [41, 42] or to provide detailed statistical analyses [40] but to map an heterogeneous body of literature related to a broad and novel topic [35]. As scoping reviews are usually considered a “richly informed starting point for further investigations” [40], future studies should consider this work as a preliminary step to a systematic review and associated statistical data analysis. Furthermore, they could use this general mapping of the health-related big data topic to generate empirically testable research hypotheses.

## Discussion

The drastic increase over the past 5 years in the number of studies discussing the implications of health-related big data confirms the research community’s increasing attention to the applicability of big data approaches into the healthcare domain. As the application of big data in healthcare [43] and the market size forecasts for big data hardware, software and professional services investments in the healthcare and pharmaceutical industry are growing steadily [44], there will be a parallel need to assess the impact of this expanding sociotechnical trend. This expansion can be seen as a sign of what has been defined the “inevitable application of big data to healthcare”[10] induced by the widespread uptake of electronic health records (EHRs), and the large-scale storing and sharing of genomic, proteomics, imaging and many other biomedical data.

The large prevalence of cross-field evaluations of health-related big data is an indicator of the potential of big data approaches to aggregate data from multiple medical data sources (e.g. combining data about gene expression and brain function in neurogenic studies) and multiple levels of clinical intervention (e.g. linking prevention and diagnostics to therapy and care delivery). In addition, analyses show that clinical outcomes can be produced from novel and non-strictly medical data sources. These include using Twitter to track and even forecast disease activity [45], exploiting Facebook data for suicide prevention [46], or using seasonal pollen forecast to predict asthma [47, 48]. On the long term, this meta-specialty nature of big data approaches is likely to blur traditional separations between different medical specialties and levels of clinical intervention, opening more interfaces for inter-specialty exchange in the healthcare and biomedical research domains. This will raise the challenge for ERCs to review big data projects without relying on traditional discrete taxonomies of medical specialization and/or models of clinical application. In parallel, our findings illustrate the potential applicability of big data approaches to an increased variety of medical specialties. While branches of medicine like oncology [49, 50], radiology [51] and clinical genetics [52] were already known to be particularly suitable for big data approaches, our review revealed a promising outlook associated with using big data in several other medical domains including neurology [53, 54], psychiatry [55], immunology [56], nephrology [57], and geriatrics [58].

The high frequency of technical challenges addressed when assessing health-related big data highlights the persistence of a number of technical weaknesses and limitations, most of which are likely dependent on the historical novelty of such sociotechnical trend. These include problems of data quality, integrity, and security. Developing robust technical solutions that can guarantee the quality, integrity and security of the data, and allow their secure transmission, linkage and storage, was often presented as a priority for any successful deployment of big data for human health. This might require the development of better security-protecting infrastructures, data wrangling and scripting (e.g. batch processing) tools for data cleansing in order to guarantee the quality of data -for example, through automatic detection and removal of corrupt or inaccurate records- as well as techniques that can preserve the integrity of data through the entire data cycle, prevent corruption and enable interoperability. Furthermore, distributed ledger technology, distributed storage and incremental analytics are also believed to hold promises in the health domain [59, 60]. From the perspective of ERCs, this implies a more rigorous yet systemic oversight [61] of technical considerations to guarantee that the afore listed safeguards are implemented by the researchers.

The relative frequency of methodological issues, however, highlights that fixing technical problems alone might not be sufficient to use big data for good. ERCs are usually required to evaluate the methodological soundness of a study if this has ethical consequences. For example, if a RCT is designed without giving participants an equal chance of being assigned to any group, ERCs are entitled to assess the methodological soundness of the study to preserve the principle of fairness. For the same reason, in the context of big data research, ERCs might be entitled to assess the soundness of studies whose methods may result in algorithmic discrimination or breaches of personal privacy. For example, they may examine whether the researchers have implemented all necessary safeguards to prevent algorithmic bias and comply with data security standards.

Examining the methodological soundness of health-related big data studies will likely require the adoption of different assessment criteria compared to traditional biomedical research. For example, it may require a rethinking of what counts as “public” data and what counts as “harm” in data-driven research. In addition, big data research is usually not based on the formulation and testing of specific research hypotheses, but on the identification of patterns from large volumes of data. This hypothesis-free nature of (some) big data research makes it harder to apply conventional epistemological mechanisms for scientific demarcation and quality control like falsifiability and refutability [62]. This poses for ERCs the problem of clearly demarcating the explanatory power of big data driven research. Researchers have questioned that big data analytics might speak for themselves [63] independent of explanatory hypotheses and refuted the idea that they can be used for biomedical purposes in absence of robust and causally explanatory scientific models or theories [64, 65].

Ethical challenges also constitute an important area of consideration for ERCs. Data breakdown by class of ethical consideration reveals that the current ethical debate is being largely monopolized by issues of privacy and data protection (Fig 3). It was already pointed out, that the ethics of big data should not be reduced to a privacy challenge but it encompasses a number of positive ethical goals [66]. Several ethical issues for which Mittelstad and Floridi [22] demanded increased ethical attention still appear largely underexplored. For example, our analysis reveals that issues of data ownership, group-level ethical harms, and the distinction between academic and commercial uses of big data, do not appear as ethical priorities. Furthermore, we observed that issues of fairness and the risk of discrimination compose a relatively small portion of the current ethical spectrum even though the misuse of big data has demonstrably resulted in various forms of ethnic, gender and class discrimination [67]. While group-level harms are usually considered outside the purview of ERCs, the dangers of ignoring this type of risk require careful assessment [68]. Issues of trust, transparency, accountability, dignity compose an even smaller fraction of the current ethical landscape. We suggest that the ethical review of health-related big data research should explore a broader spectrum of ethical issues. In particular, it should scrutinize more carefully (i) whether and how each project attempts to address the social benefits, if any, of research; (ii) how data subjects involved in the study can exercise control over their data (data control problem); (iii) which measures of accountability are being employed by the researchers, (iv) whether the collected data can be reused for secondary, including malevolent, purposes (dual use problem) and what measures are implemented to prevent that.

These technical, methodological and ethical challenges should not be seen as sealed rooms. Thematic analysis reveals an intimate interconnection between the three thematic families. For example, the technical problem of data security appears strictly connected to the ethical notion of privacy and the regulatory principle of data protection. Similarly, methodological errors like dataset bias might have detrimental ethical consequences such as racial and gender discrimination. This intimate link between technical and ethical issues highlights the importance of cooperative approaches to study design in big data research through strategies like ethical design of data-collecting technologies, proactive ethical assessment of big data studies and ethical requirement analyses for data-sharing platforms, data storage sites and other digital infrastructures. ERCs should be sensitized to this interconnection and examine how weaknesses in one domain affect other domains of evaluation. Similarly, the interdependence of epistemological and ethical issues, which was already highlighted by Mittelstad and Floridi [22], requires careful consideration by ERCs to prevent that inaccurate study designs or data curation practices result in unintended harms to individuals or groups.

Overall, these findings have three main and direct implications for ERCs. First, the significance and complexity of technical and methodological challenges suggests that members of ERCs should need to acquire stronger technical and methodological expertise to adequately review and evaluate health-related big data studies. This might require specific educational courses or other training activities aimed at strengthening ERC-members’ ability to identify technical/methodological problems or inaccuracies, especially those that can result in harms to data subjects or society like data security breaches, database corruption and biased algorithm training. Specialized training modules in data science, bioinformatics and cybersecurity might serve this purpose. In parallel, as emerging from the normative suggestions, ERCs need to consider including experts from the afore listed disciplines within the review board. Since health-related big data is here to stay, new expert profiles are needed during the review process. Data scientists, security experts, bioinformaticians should complement the expertise of clinicians, ethicists and other traditional ERC members. ERC members will need to be equipped with the necessary tools to inspect how the data will be collected, in conformity with which security standards they will be stored and shared, what classification systems will be employed, how uncertainty will be quantified, what cluster models will be adopted during exploratory data mining etc.

In spite of these important challenges, ERCs might still be faced with uncertainty when reviewing health-related big data studies. Review results indicate that only a tiny fraction of studies (13%) provided specific normative recommendations for ERCs. These are suggestions or proposals for ERCs as to the best course of action. Further thematic analysis reveals a general disagreement and a lack of consensus on what codes of conduct should be prioritized, with some authors [25] favouring the simplification of the ethics review process and others [69] requiring more stringent scrutiny. Nonetheless, five recurring themes could be identified: (i) preventing the dangers of downstream data linkage and inadvertent individual identification; (ii) expanding the purview and involvement of ERCs; (iii) developing a clearer understanding of the risks and benefits of health-related big data research, (iv) harmonizing ethical standards for big data research and (v) rethinking the composition of ERCs. The extremely small fraction of studies providing normative recommendations informed by empirical research (i.e. based on studies involving direct observation or experience such as survey questionnaires or focus groups), further underscores how these recommendations are mostly based on individual viewpoints rather than on solid consensus within the research community.

In the debate on what ERCs should do in relation to health-related big data, the opinion of ERC members is missing. Future empirical research is highly required to explore the needs, views and attitudes of ERC members about health-related big data. Empirical research in this domain could methodologically build upon previous studies involving ethics advisors working in big-data-related areas of research such as genomics governance [70]. Combining empirical and normative ethical research in the health-related big data domain would not only benefit the understanding of the current problems that ERCs are facing when reviewing health-related big data studies, but also favour the development of empirically-informed research ethics guidelines [71], hence resulting in better ethical oversight and governance of the health-related big data phenomenon.

Finally, it is legitimate to raise the question of whether ERCs should be the only governance body responsible for the evaluation of biomedical big data research. Given their traditional mandate, which is deeply rooted in the pre-digital era of biomedical research, it might be reasonably argued that ERCs are ill-suited to exercise exclusive ethical oversight on health-related big data research. Research regulators should consider whether complementary governance mechanisms such as data boards, data security committees or allied bodies are necessary to expand the bandwidth and sensitivity of ethical oversight.

## Supporting information

S1 File Search strategy. (DOCX)

S2 File Dataset. (XLSX)

S3 File Compressed article repository. (ENLX)
