# Supplementary material for: Considerations for ethics review of big data health research: A scoping review
Source: PLoS One. 2018 Oct 11;13(10):e0204937. doi: 10.1371/journal.pone.0204937 (PMC6181558; doi:10.1371/journal.pone.0204937)
Supplement: S1 File — (DOCX) [file pone.0204937.s001.docx]

**Search Strategy**

Search performed on search engine: Web of Science

Time range: until September 18, 2017.

You searched for: Title/Abstract: (medical OR healthcare OR clinical OR "personalised medicine") AND Title/Abstract:("big data" OR “Artificial Intelligence” OR "data science" OR "digital data") AND Title/Abstract: (policy OR ethics OR governance OR "ethics committee" OR “IRB” OR "review board" OR assessment)
